# Linking Authentic Leadership to Transactive Memory System, Team Innovativeness, and Selling Performance: A Multilevel Investigation

**DOI:** 10.3389/fpsyg.2022.884198

**Published:** 2022-07-22

**Authors:** Muhammad Asim Shahzad, Tahir Iqbal, Muhammad Imad Ud Din Akbar, Khuda Bakhsh, Bilal Ahmad

**Affiliations:** ^1^School of Economics and Management, North China Electric Power University, Beijing, China; ^2^Department of Business Administration, University of Zaragoza, Zaragoza, Spain; ^3^Department of Management Sciences, National University of Modern Languages, Lahore, Pakistan; ^4^Department of Education, GC University Faisalabad, Faisalabad, Pakistan; ^5^Riphah School of Business and Management, Riphah International University, Lahore, Pakistan

**Keywords:** authentic sales leadership, transactive memory system, innovative work behavior, customer-directed OCB, team selling performance

## Abstract

In today’s complex selling environment, it is challenging for sales leaders to enhance the effectiveness of their sales teams. The aim of this study is to observe the impact of authentic leadership on salespersons’ internal and external behaviors under B2B selling context [i.e., transactive memory system (TMS), innovative work behavior, and customer-directed OCB] and their consequences in team selling performance. Respondents of our survey included salespersons and managers working in the sales departments of pharmaceutical companies. By using structural equation modeling, the dyad responses from 348 matched salespeople–managers were analyzed. The findings disclose that authentic leadership behavior has a stronger relationship with the TMS, innovative work behavior, and customer-directed OCB. Our results also indicate that innovative work behavior and customer-directed OCB are potentially mediated between authentic leadership and team selling performance relationship. The theoretical implication of these results for managerial practice is also discussed.

## Introduction

In most industries, the sales cycle is getting longer and more complex (Plouffe et al., 2017). As customer complexity increases, sales and marketing leaders should constantly regulate the shifting eventualities of industry. Many scholars have said that authentic leadership behavior promotes continuous effects in social behaviors such as highly principled and ethical values (Gardner et al., 2005; Joo and Jo, 2017). Authentic leaders have major resources such as self-knowledge, clarity of self-impression, and self-image values (Gardner et al., 2005), which motivate leaders to act as a resource for social support to followers’ internal and external behaviors at a personal level and subordinates at work-unit level (Zhou et al., 2014; Braun and Nieberle, 2017). However, this multilevel perspective encouraged authors to adopt the conservations of resources (COR) theory (Hobfoll, 1989) to address the perception of authentic leadership as a resource in organizations at different levels (i.e., team level and individual level) as well as its consequences on overall team performance. Authentic leaders aim to help salespeople to develop their resource pool as a source of motivation for subordinates (Braun and Nieberle, 2017). They could engage in different sales activities beyond minimum requirements, such as innovative work behavior and customer-directed citizenship behavior. This study can place authentic leadership as a unique style to the success of sales employees from the resource-based perspective, which is parallel to more commonly studied leadership styles in sales literature.

Besides, the conservation of resources (COR) theory also facilitates implementing a transactive memory system (TMS), which can be used as a resource to invest in team development. Thus we can argue that TMS might affect team-level performance. Multilevel sales departments allow cross-group efforts and the circulation of job-related tasks according to the area of capability. In this regard, certifying a well-designed TMS is specifically important among all groups (Faraj and Yan, 2009; Kotlarsky et al., 2015). A TMS is described as a joint department of team members to acquire, understand and transmit team-related information (Hollingshead, 2001; Yan et al., 2021; O’Toole et al., 2022). Since the TMS is a possible antecedent in sales literature, the connection between authentic sales leadership and TMS has not been researched or examined. These two concepts have seemed to be explored in two separate studies (Hollingshead, 2001; Gardner et al., 2005). Analyzing the influence of authentic sales leadership through the COR perspective indicates that a comprehensive strategy for resource gain should be a process that ties genuine sales leadership to a TMS.

Innovative work behavior is valuable for both organizational objectives and team selling performance. Previous work has witnessed innovative work behavior as the mediating mechanism (Buranakul et al., 2017; Sanz-Valle and Jiménez-Jiménez, 2018). However, the current research differentiates this constructive mediation mechanism from previous leadership and job performance studies, such as job fulfillment, organizational loyalty, perceived emotional well-being, and work engagement (Ashill et al., 2008; Guchait et al., 2014; Karatepe and Olugbade, 2016). Additionally, to explore authentic leadership effect through the COR perspective advocates that a comprehensive strategy for resource gain should be the procedure of relationship with customer-directed OCB (Luu, 2020).

To sum up, this research covers three important gaps in the B2B sales leadership literature. First, the research is the earliest attempt to explore the effect of authentic leadership as a resource to the TMS that exchange and retrieve useful knowledge among workgroups. Our study looks into team selling performance that the authentic leadership literature has mostly ignored in the B2B sales context. The related outcomes in authentic leadership literature involved employee innovation and job performance (Wang et al., 2014; Zhou et al., 2014). Second, the examination of authentic leadership findings through the viewpoint of COR theory proposes that a constructive resource benefit method could connect authentic leadership to the employee’s extra-role behavior toward customers. However, to the best of the authors’ understanding, only a few trials have been performed on the connection between authentic sales leadership and customer-directed OCB. As a result, we are attempting to address this void in this research. Third, previous innovation research has relied extensively on the consequences and mediating mechanisms of innovative work behavior (Riaz et al., 2018), while overlooking the interactional effect of employees’ innovation with any other discretionary behavior. Customer-directed OCB has previously been viewed as an important discretionary behavior (MacKenzie et al., 1999), and classifies the salespeople who go beyond and above the call of duty for consumers. Our research explores how innovative work behavior in a combination of customer-directed OCB influences sales team performance. Additionally, the research also examined innovative work behavior as a potential mediator between authentic sales leadership and team selling performance relationships. By visualizing this argument, we claim that this research significantly adds value to the growing body of literature.

## Theoretical Background and Hypotheses

Many authors note the importance of the principle “to be true of yourself,” which has become a major part of authentic leadership theory. Although in order to be genuine, we sometimes ignore that one must also be truthful to others. Due to the increased number of corporate scandals, dishonesty, and unethical activities undertaken by business leaders, authentic leadership has gained empirical popularity over time (Gardner et al., 2011). In the pharmaceutical industry, it has been deemed necessary among scholars and practitioners to put their analytical lens on this leadership style. However, authentic leadership which tends to be important for team selling performance may provide a unique concept to support other sales leadership frameworks. According to COR perspective, the study sheds light on how authentic leadership affects salespersons’ internal and external sales behaviors at different levels within the organizations.

### Authentic Leadership and Transactive Memory System

In the context of improving team-level consequences, one of the prime goals of this research is to develop insights into authentic leadership and TMS in B2B selling context. The COR theory proposes that individuals always attempt to gain, preserve, defend and encourage various forms of resources (Hobfoll, 2001). In this context, TMS can be viewed as a valuable enterprise resource for individual salespeople because it provides a friendly and knowledge-exchange climate that overcomes mental stress and improve teamwork to accomplish tasks. According to COR theory, people need to spend resources at work (i.e., TMS) on the development of skills or competencies and enforce against the possible loss of resources or acquire more resources (Hobfoll et al., 2018).

Additionally, authentic leaders are observed as authentic (i.e., responsible, genuine, and honest) by followers. The decision-making of authentic leaders is transparent and associates well with their subordinates (Avolio et al., 2009). The TMS provides critical information that enables coworkers to easily exchange their knowledge. Besides, the team with a well-designed TMS will share knowledge more efficiently. Previous research highlights the relationship of authentic leadership with expertise shared by the followers (Reed et al., 2011). In a study, Peterson et al. (2012) suggest that authentic leaders should encourage their followers to trust the working environment and be able to retrieve and share their knowledge with other colleagues in order to establish trust. It is stated by Hahm (2017) that followers who are influenced by authentic leaders will have a tendency to retrieve and share their specified knowledge and capabilities with other colleagues for overall team achievements. Therefore, we suppose that authentic sales leadership may have a positive influence on the TMS and suggest the following hypothesis.

*H1*: Authentic sales leadership is positively related to the TMS.

### Authentic Sales Leadership and Innovative Work Behavior

An important variable in our research model is innovative work behavior. Innovative work behavior can be outlined as a salesperson’s purposeful impression of unique ideas, products, procedures, and practices in his/her working environment (Esam et al., 2012). The COR theory suggests that innovative work behavior is now one of the aspects in which salespeople could improve or decline as a means of acquiring or maintaining valuable resources (Kiazad et al., 2014). According to authentic leadership theory, authentic leaders can support innovation by encouraging their team members to be more brave and creative (Avolio et al., 2004). Organizational creativity literature suggests that leaders and corporations should develop a positive workplace environment for improving employee innovative work behavior. Authentic leaders have the ability to develop healthy emotions in their team members by fostering optimistic, supportive, and fair relationships, which results in increased innovation (Peterson et al., 2012). Prior pieces of evidence have proved an association between ethical observation and employee innovative work behavior (Bierly et al., 2009). According to Walumbwa et al. (2008), authentic leadership dimensions (self-awareness, internalized moral perspective, relational transparency, and balanced processing) encourage innovativeness. For example, relational transparency is responsible for innovation by expressing new ideas, difficulties and transmitting useful information explicitly. Therefore, based on the aforementioned argument we suggest the following hypothesis.

*H2*: Authentic sales leadership is positively related to innovative work behavior.

### Authentic Sales Leadership and Customer-Directed OCB

To inspire and support subordinates, authentic leaders frequently exchange resources for making decisions if necessary and are conscious of their personal opinions, standards, objectives, and emotions (Wang et al., 2014). Authentic leaders can inspire their subordinates through a reflective form of commitment over a longer duration to produce effective results (George, 2003). Furthermore, with customer-directed OCB salespeople may serve and solve the customer problems by going out of their roles and assigned duties, such as fulfilling customer’s expectations, user-friendly services, or discovering an appropriate way to expand the customer delivery process. However, in consistent with a recent study on authentic leadership (e.g., Braun and Nieberle, 2017), we take into consideration the COR theory (Hobfoll, 1989) just to highlight that how authentic leadership influences their sales team’s productivity in terms of delivering consumers by going beyond and above their assigned duties (Luu, 2020). According to COR viewpoint, “gaining sufficient resources from a source of resources, individuals are inclined to take a positive, rather than defensive, resource gain strategy to increase additional resources and spend their behaviors above and beyond the minimum expectations” (Halbesleben et al., 2014). Peus et al. (2012) have suggested that authentic leadership could be viewed as a possible predictor of employee extra-role behavior. Salespeople are encouraged to devote their time and energy to customer-directed OCB by retrieving resources from authentic sales leaders and acknowledging the fundamentals of those sales-related activities for themselves. We have thus formulated the following hypothesis based on the argument mentioned earlier.

*H3*: Authentic sales leadership is positively related to customer-directed OCB.

### Transactive Memory System, Innovative Work Behavior, and Team Selling Performance

Transactive memory systems allow a group member to communicate with other teams, to set their plans more wisely. The most capable member of the team should be preferred for assigning tasks and to support teams to resolve the problem more speedily (Liang et al., 1995). This might be realistic to say that a TMS would have an impact on team-level inventions and outcomes, which is an evolving benefit (Fan et al., 2016). Field studies on executive teams have claimed TMSs as a facilitator of team overall success (Faraj and Sproill, 2000; Lewis, 2004). Despite this, numerous studies have emphasized the concern that the essential TMS-team improvement framework is still unclear, and it has multiple mediating paths such as team productivity (Dayan and Di Benedetto, 2009), team reflectiveness (Dayan and Basarir, 2010), team effectiveness (Zhong et al., 2012). In a performance context, where the TMS is considered relevant, the innovative work behavior tends to be consistently involved in problem-seeking and problem-solving activities such as searching for unique and effective ideas. Therefore, we predict that in a team-based situation, TMSs replicate two dimensions of Amabile (1996) model, which eventually impact salespeople’s innovative work behavior. When employees work in a fully advanced TMSs environment, the team communicates valuable information about the actual findings of work-related activities, allowing them to demonstrate a high degree of meaningful engagement and establish new work patterns. Therefore, we hypothesize that individuals are enthusiastic about working innovatively and enjoying their responsibilities more when engaged in high TMSs. Thus, we propose the following hypotheses.

*H4a*: Transactive memory system is positively related to innovative work behavior.

*H4b*: Transactive memory system is positively related to team selling performance.

### Customer-Directed OCB, Innovative Work Behavior, and Team Selling Performance

**Figure 1:**
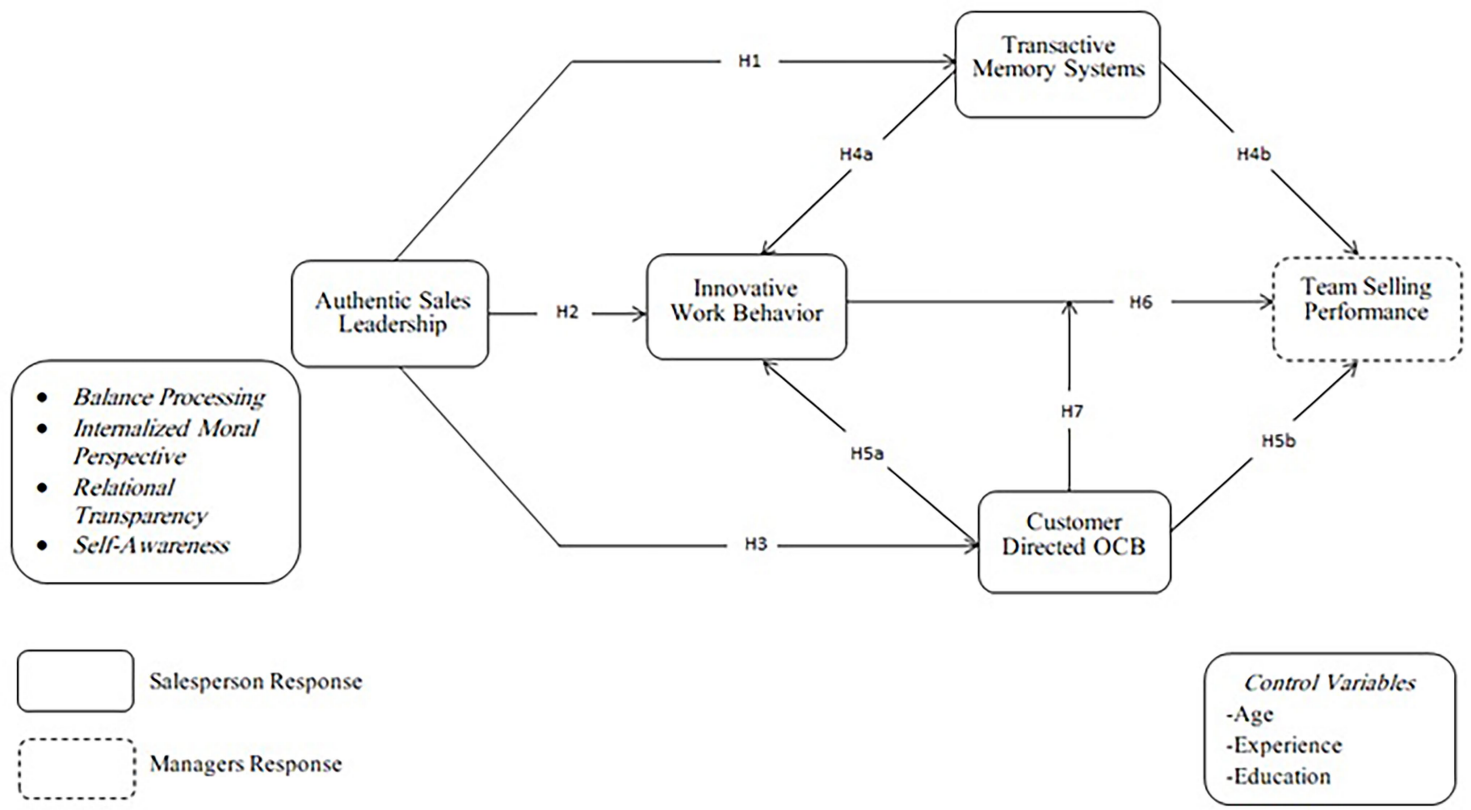
Conceptual framework.

Customer-directed OCB is perceived as an employees’ unauthorized behavior when serving customers outside of the formal job responsibilities (Moliner et al., 2008). This action creates a sense of appreciation, encouraging the customer to support the salesperson as the salesperson goes out of the work to support or reward the customers. If leaders are very innovative, an employee seems to be more optimistic in the team’s innovative activities. As a result, employees are encouraged to stick with the plans when faced with obstacles and make a strong initiative for the team whenever they want to accomplish shared goals (Deng and Guan, 2017). Subordinates may take part in more productive behavior that benefits both the company and the consumers, which can be defined as their citizenship behaviors. This study is contextualized in the pharmaceutical context. We know that pharmaceutical salespeople interact with extremely well-informed practitioners (i.e., physicians, clinicians, and pharmacists). However, there is hardly any clear connection between a visit by salespersons to the general physician (GPs) and the purchase of drugs. Consequently, pharmaceuticals are not normal products even physicians are very odd customers; so that it is hard for salespeople to manage their expectations and to satisfy their needs. Social exchange theory proposes that a customer would only regard the efforts of sales employees when they are fully committed to their word of mouth promotion and additional businesses (Podsakoff and MacKenzie, 1997). It is stated by Miao and Wang (2016), when salespeople engage in customer-directed extra-role behavior, the customer would not only be willing to adopt the innovative solution by salespeople, but it helps them to turn innovativeness into overall team selling performance. Hence we proposed the following hypothesis (see Figure 1).

*H5a*: Customer-directed OCB is positively related to innovative work behavior.

*H5b*: Customer-directed OCB is positively related to team selling performance.

### Innovative Work Behavior and Team Selling Performance

Innovative work behavior involves salespeople who exhibit distinct behaviors in terms of personal gains and innovative ideas related to team effectiveness (Jiménez-Jiménez and Sanz-Valle, 2011). Previous studies have been investigated that employees’ innovative work behavior enhances team-level performance (Oldham and Cummings, 1996). Innovative behavior is also considered as creative behavior, and the purpose of this behavior is not only to produce new ideas by oneself but also to adopt other ideas that are new or unique to other team members and units (Woodman et al., 1993). Also, salespeople’s innovative behavior comprises both the creation and execution of novel ideas (Shalley et al., 2004). Sales team innovation and creativity are valuable to achieve a firm’s objective and sales performance. Further, innovative work behavior is considered sometimes risky and beyond the job responsibility by salespeople. So, sales managers must provide a suitable environment of trust, belief, and support to these innovative traits (Anderson et al., 2004). Thus, focusing on the foregoing discussion we proposed the following hypothesis.

*H6*: Innovative work behavior is positively related to team selling performance.

### The Moderating Role of Customer-Directed OCB

Luu (2020) classified customer-directed OCB as salespeople working out of the way or across the call of duties for customers. When salespeople engage in customer-directed organizational citizenship behaviors, they provide a high quality of customer experiences. Therefore customers often likely to adopt the salesperson’s creative solutions, which directly impact overall team sales performance. Social exchange theory (Cropanzano and Mitchell, 2005) is significant to customer service experiences in which customers and sales service providers have reciprocal expectations. The role of citizenship behavior toward employees’ innovativeness has been identified as advantageous (Kesen, 2016). Innovative workers must explore and encourage innovations and find resources for their execution (Amabile, 1988). If teams play their role well then leaders expect team members to expand their existing performance and survive in the long tenure. Many studies have been conducted with the moderating role of employees’ citizenship behavior, such as between engagement and employee retention relationship (Farooq, 2015), and employee commitment and performance relationship (Hakim and Fernandes, 2017). However, it is hard to find the moderating role of customer-directed OCB between the linkage of innovative work behaviors and team-level sales performance. In light of the above argument, we may assume that the connection among innovative work behavior and team selling performance will be stronger with the interaction effect of customer-directed OCB.

*H7*: Customer-directed OCB significantly moderate among the linkage of innovative work behavior and team selling performance such that this relationship is stronger with the greater level of customer-directed OCB.

### The Mediating Role of Transactive Memory System, Innovative Work Behavior, and Customer-Directed OCB

Several studies demonstrate a TMS to be vital in improving team performance at all levels (Kotlarsky et al., 2015; Cao and Ali, 2018). However, limited inquiries have been performed on the TMS in the mediation relationship (Fan et al., 2016). A TMS defines a collaborative team network where participants typically establish to collectively acquire and retain knowledge and expertise in various disciplines (Lewis and Herndon, 2011). Previous research has claimed that the followers’ expertise enhances collaboration among the teams under authentic leadership (Reed et al., 2011). According to Hahm (2017), when authentic leaders influence the followers, they improve the tendency to share specialized expertise and useful information with other colleagues for overall team achievement. Meanwhile, prior field studies on structural teams have also claimed TMSs as a supporter of team-level performance (Faraj and Sproill, 2000; Lewis, 2004).

It has been proven that many large sales organizations develop and flourish in the long term just because of their innovative sales employees (Amabile, 1988). Authentic leadership theory suggests that authentic leaders can support innovation by motivating their subordinates to be more creative and enthusiastic (Avolio et al., 2004). It is mentioned in organizational creativity literature that leaders and corporations should develop a positive environment in the workplace for improving employees’ innovative work behavior. The positive emotions of salespeople could be improved under authentic sales leadership by creating positive, original, and fair relations, which turns into more innovation. Prior literature suggests that salespeople’s innovative work behavior enhances team performance, and their creativity and innovation are meant to achieve firms’ overall objectives (Oldham and Cummings, 1996). Many researchers have been investigated the mediating role of innovative work behavior among different constructs (Buranakul et al., 2017; Sanz-Valle and Jiménez-Jiménez, 2018). However, the literature has neglected to explore the mediating effect of innovative work behavior between the nexus of authentic leadership and team selling performance. Furthermore, many pieces of evidence have been gathered on the positive influence of authentic leadership on team selling performance (Wong and Laschinger, 2013; Luu, 2020). Thus, the above argument supports the mediating effect of innovative work behavior between the above relationships.

In addition, the current study formulates the COR theory to link authentic sales leadership with customer-directed OCB. This is particularly appropriate for authentic leadership behavior because it provides a transparent and competitive work atmosphere that has a direct impact on employee behaviors, provides a high degree of wellbeing, faith, and motivation to implement extra-role duties (Avolio et al., 2004; Avolio and Gardner, 2005). It is pointed out by MacKenzie et al. (1999) that customer-directed OCB accounts for a higher level of the intervention of salesperson performance. Hence, strong evidence allows us to predict the mediating role of customer-directed OCB among the nexus of authentic leadership and team selling performance. We, therefore suggest the primary hypotheses focused on the above conversation.

*H8a*: Transactive memory system is not significantly mediating the relationship between authentic sales leadership and team selling performance.

*H8b*: Innovative work behavior is significantly mediating the relationship between authentic sales leadership and team selling performance.

*H8c*: Customer-directed OCB is significantly mediating the relationship between authentic sales leadership and team selling performance.

## Materials and Methods

### Survey Sample and Data Collection

The model was tested by collecting multilevel data set, including matched surveys from managers and salespersons. A cross-sectional survey method was performed to collect the data. We approached the majority of the sales managers from different pharmaceutical companies in Pakistan. To measure the selling performance of team members, we requested the team managers to evaluate individual performance separately to avoid the common method biased. This situation is considered perfect for analyzing our framework because selling performance is based on the capability of sales team members to offer customized services to accomplish the specific requirements of customers. Importantly, individuals of the sales teams worked collaboratively for information sharing, encouraging and empowering each other to clarify customer responsibilities. Firstly, we needed to get approval and assistance from each pharmaceutical company’s management for collecting the data. We decided 86 pharmaceutical companies to participate in our survey, in which 52 companies accepted our invitation. We then gathered relevant information of 105 team sales managers from each company’s HR department and contacted them physically and telephonically to participate in our online survey. We administered 20 thoroughly qualitative interviews with sales managers and sales team members before collecting the data to ensure the authenticity of the survey material. Later on, one manager and one to four salespeople were randomly selected to conduct the survey. An online questionnaire link was therefore mailed to the sales managers and requested them to forward the link to each team member. We also asked the salespeople to enter a five-digit number and give it back to their respective managers in order to match their responses from both managers and salespeople. We distributed survey questionnaires to 420 salespeople and their 105 respective team sales managers. After the survey completion, we removed the sales teams with less than four responses from sales employees. The final matched sample resulted in 348 valid responses from salespeople and 87 valid responses from sales managers, yielding 348 sales manager-salesperson dyads. There were 73.5% men among the survey participants. The participants were also qualified; 66.2% had received their 4 year of graduation degree. In terms of experience, 62.3% of respondents had worked in sales for more than 10 years in different organizations.

### Construct Measures

We designed a questionnaire to evaluate the hypotheses. The measuring factors have been modified from the prior studies. All the components were assessed on 5-point Likert scales ranging from “1 = strongly disagree” to “5 = strongly agree.” To measure *Authentic sales leadership*, we adapted 13 items scale measuring four dimensions (self-awareness, balanced processing, relational tendency, internalized moral perspective) from previous literature by Walumbwa et al. (2008). A sample item is “My manager is aware of what he truly finds important.” *Transactive memory system* was adapted from previous literature by Lewis (2003), and it has been measured on a 9 items scale. A sample item is “Each team member has specialized knowledge of some aspect of our project.” *Innovative work behavior* was adapted from the study of Scott and Bruce (1994). The responses were assessed on a three-item scale. A sample item is “I create innovative solutions for problems.” *Customer-directed OCB* was also adapted from the previous study of Miao and Wang (2016) and measured on a 4 items scale that responded by ‘1 = Never to ‘5 = Very frequently. A sample item is “I work more than my duty when serving customers.” *Team selling performance* is adapted from the studies of Singh and Das (2013) and Itani et al. (2017) and measured on 4 items scale. A sample item is “My sales team goes above the sales targets.”

## Analysis and Results

### Model Specification Testing

The current research contains multiple dependent and independent variables; we used structural equation modeling (SEM) by using SmartPLS software. This approach helps researchers to test theoretical questions, for instance, we describe in the model specifications. The growing use of PLS-SEM has demonstrated its robustness and the applicability of the model in the area that is being studied. Our structural model is diverse and requires multiple structures, which encouraged authors to use PLS-SEM. Table 1 describes the correlation among variables. The correlation values are lower than the standard value of 0.65 (Tabachnick, 1996; Heavey and Simsek, 2015), and the maximum variance inflation factor VIF (2.79) is below the threshold of 3.3 (Kock, 2015), thus proposing that the multicollinearity is not a problem.

**Table 1:** Correlation matrix.

S. No	Variable	1	2	3	4	5	6	7	8
1	Authentic leadership	(0.756)							
2	Customer directed OCB	0.365^**^	(0.718)						
3	Innovative work behavior	0.629^**^	0.310^**^	(0.548)					
4	Team selling performance	0.109^*^	0.121^*^	0.109^*^	(0.622)				
5	Transactive memory system	0.167^**^	0.121^**^	0.185^**^	0.119^*^	(0.686)			
6	Age	0.144^**^	−0.012	−0.075	0.124^*^	0.056			
7	Experience	0.099	−0.116	−0.025	0.182^**^	0.020	0.120^*^		
8	Education	−0.108	−0.600	−0.088	0.080	0.094	−0.045	−0.051	

*N* = 365; OCB, organizational citizenship behavior.

* Correlation is significant at the 0.05 level, *p* < 0.05.

** Correlation is significant at the 0.01 level, *p* < 0.01.

To measure the validity and reliability of the variables, we conducted a multi-factor analysis (Gerbing and Anderson, 1988). The coefficient values of reliability are as follows: 0.91 for authentic sales leadership, 0.88 for the TMS, 0.76 for innovative work behavior, 0.72 for customer-directed OCB, and 0.81 for team selling performance. In Table 2, we conducted confirmatory factor analysis, and the results of each loading item in the conceptual model were exceeding the projected value of 0.50 (Arbuckle, 2016). Consequently, we acknowledge the importance of each measure to the developed variable. The average variance extracted and composite reliability surpasses the suggested standard value of 0.50 (Fornell and Larcker, 1981). Then we prove the discriminant validity even by the assumption that the average variance extracted of each variable must exceed the squared correlation within each group of variables (Fornell and Larcker, 1981). The results of standardized root mean square residual (SRMR) = 0.06; normed index fit (NIF) = 0.302 and chi-square (*X^2^*) =837.253 shows the suitable fitness of measurement model.

**Table 2:** Factor loadings of items.

Variables	Constructs/items	Standard	Alpha	CR	AVE
		Loading	*α*		
*Authentic leadership (aggregated from individual level)*					
(Balanced processing)			0.91	0.92	0.58
When someone criticizes my manager, he tries not to pay too much importance to it.		0.803			
My manager would rather not have individual weaknesses to be revealed.		0.784			
My manager tries to block out annoying feelings about himself.		0.774			
*(Internalized moral perspective)*					
My manager stays true to his personal values.		0.636			
Individuals can rely on my manager to behave in the same way over situations.		0.619			
My manager acts according to personal values, even if others find fault with him for it.		0.546			
*(Relational transparency)*					
My manager often pretends to like something when he does not.		0.721			
Even when my manager disagrees with somebody, he will often quietly reach an agreement.		0.879			
My manager often behaves in a way that does not replicate his true feelings or thoughts.		0.784			
My manager often pretends to be someone he is not.		0.741			
*(Self-Awareness)*					
My manager is aware of why he does the things he does.		0.692			
My manager is aware of what demotivates him.		0.711			
My manager is aware of what he truly finds important.		0.732			
Transactive Memory System (aggregated from individual level)			0.88	0.89	0.53
*(Coordination)*					
Our team worked jointly in a well-coordinated style.		0.892			
Our team had very rare confusion about what to do.		0.762			
We accomplished the task effortlessly and professionally.		0.780			
*(Credibility)*					
I was comfortable accepting practical recommendations from other fellow workers		0.703			
I trusted that other members’ expertise about the task was reliable.		0.767			
I was self-assured in trusting the information that other team members brought to the discussion.		0.645			
*(Specialization)*					
Each team member has specific knowledge of some aspect of our project.		0.773			
Different fellow workers are accountable for expertise in different sectors.					
The specified knowledge of different team members was required to complete the project achievable.					
*Innovative Work Behavior (captured from individual level)*			0.76	0.69	0.51
I create innovative solutions for problems		0.194			
I invent new methods to perform tasks		0.812			
I make significant organizational members passionate for innovative ideas		0.701			
*Customer-directed OCB (captured from individual level)*			0.72	0.81	0.52
I work more than my duty when serving customers.		0.578			
I make my customer satisfied by going out of the way		0.806			
As on customer demand, I support them even if it is going more than my job requirements.		0.723			
I always help customers with problems.		0.771			
*Team Selling Performance (captured from managers)*			0.81	0.86	0.55
My sales team goes above the sales targets.		0.438			
My sales team generates a high level of sales.		0.620			
My sales team sells a full range of products.		0.746			
The sales of my team are compared to the top-performing sales group in the company.		0.346			

*N* = 365; CR, composite reliability; AVE, average variance extracted; OCB, organizational citizenship behavior; Items are measured on five-point Likert scale, where 1 = strongly disagree and 5 = strongly disagree.

### Common Method Variance

To evaluate the common method variance (CMV) firstly, we conducted Harman’s one-factor test (Podsakoff et al., 2003). Harman’s one-factor experiment allows all measurements to be loaded into exploratory factor analysis, assuming that a single factor is accountable for most covariance. By using SPSS 22, we performed a factor analysis of all indicators used in the model. The outcomes disclosed that the total explained variance of a common factor is 31.56%, indicating that common method bias in our research is not the main trouble. Furthermore, we adopted a method suggested by Kock (2015) in SmartPLS 3 to assess the CMV. According to this method, if the variance of VIF is larger than 3.3, then it is the signal that the framework is treated with CMV. The study shows the factor level VIF value lower than the recommended threshold 3.3, considering the model is excluded from CMV.

### Aggregating Data Into Team-Level Measures

To aggregate our response results to the team level, we evaluated inside and between-group variance and rater reliability components. The appropriateness of group-level aggregation of member scores was inspected by intra-class correlation [i.e., ICC(1) and ICC(2)] and inter-rater agreement index (Stewart et al., 2005). The ICC(1) measures the proportion of variation due to group participation. In contrast, the ICC(2) demonstrates the reliability of a group’s means (Hox, 2002). The inter-rater agreement and average ICCs for authentic sales leadership were *r*_wg(*j*)_ = 0.94; ICC(1) = 0.41; and ICC(2) = 0.87. For TMS we found a mean *r*_wg(*j*)_ = 0.88; ICC(1) = 0.46; and ICC(2) = 0.77. Consequently, the average values of team selling performance were *r*_wg(*j*)_ = 0.90; ICC(1) = 0.38; and ICC(2) = 0.74. Prior studies have suggested that a value of 0.70 or above is observed as satisfactory in terms of ICC(2) and within-group inter-rater agreement (Biemann et al., 2012), which demonstrates the data suitability for the study at a team level.

### Coefficient of Determination (*R*^2^)

**Figure 2:**
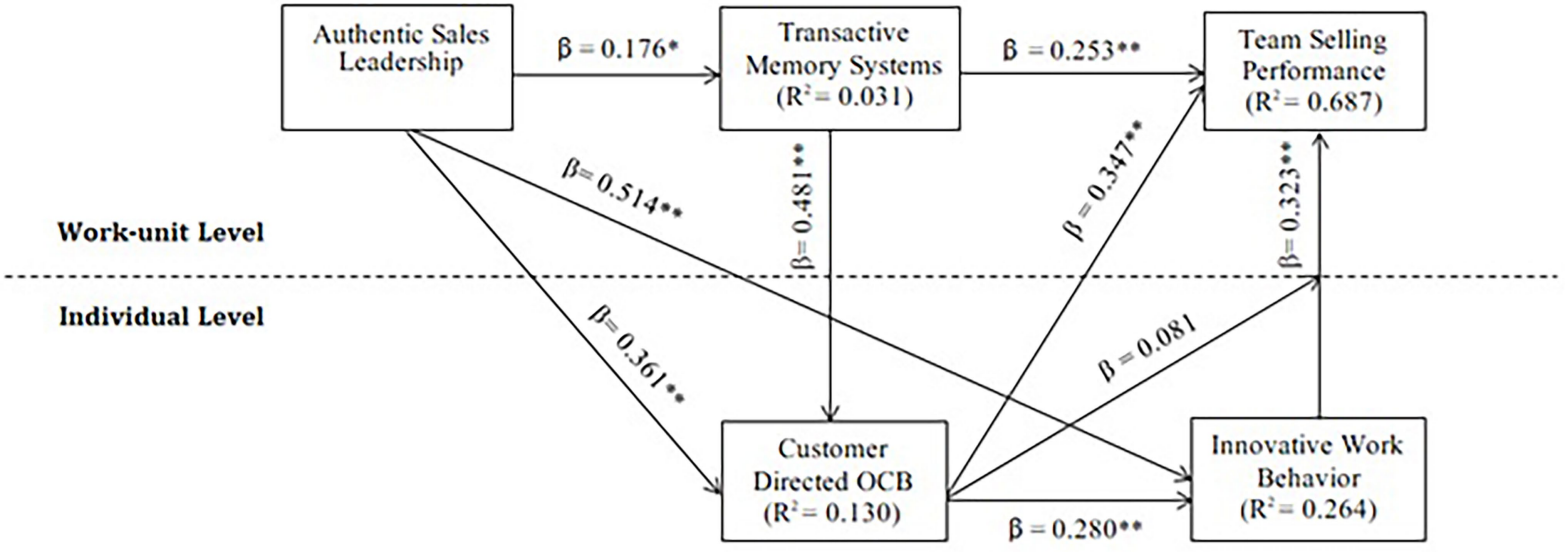
Results of hypotheses.

In Figure 2, our results show that the predictor variables explain 68.7% (*R*^2^ = 0.687) of team selling performance variance. In addition, authentic sales leadership explains 3.1% of TMS (*R*^2^ = 0.031), 26.4% of innovative work behavior (*R*^2^ = 0.264), 13% of customer-directed OCB (R^2^ = 0.130).

### Effect Size (*f*^2^)

We carried out multiple PLS estimation, each time eliminating a dominant variable in our conceptual model just to classify the influence of exogenous variable on endogenous variable. It is suggested by Cohen (1988), *f*^2^ values of 0.02, 0.15, and 0.35 are considered small, medium, and large, respectively. Table 3 shows that authentic sales leadership has large effects on innovative work behavior (*f*^2^ = 0.359), while it has small effects on both TMS (*f*^2^ = 0.032) and customer-directed OCB (*f*^2^ = 0.149). In addition, TMS has medium effects on innovative work behavior (*f*^2^ = 0.226) and team selling performance (*f*^2^ = 0.215). Similarly, customer-directed OCB has also medium effects on both innovative work behavior (*f*^2^ = 0.282) and on team selling performance (*f*^2^ = 0.192).

**Table 3:** Effect size (*f*^2^) statistics for the general model.

Hypotheses		*f* ^2^	Effect
H1	Authentic leadership → Transactive memory system	0.032	Small
H2	Authentic leadership → Innovative work behavior	0.359	Large
H3	Authentic leadership → Customer directed OCB	0.149	Small
H4a	Transactive memory system → Innovative work behavior	0.226	Medium
H4b	Transactive memory system → Team selling performance	0.215	Medium
H5a	Customer directed OCB → Innovative work behavior	0.282	Medium
H5b	Customer directed OCB → Team selling performance	0.192	Medium
H6	Innovative work behavior → Team selling performance	0.175	Medium

*N* = 365; OCB, organizational citizenship behavior.

### Predictive Relevance (*Q*^2^)

To evaluate the predictive relevance of our framework, we performed a Stone and Geisser test by using the blindfolding method on SmartPLS. It is proposed by Hair et al. (2016), that a model contains predictive relevance if the *Q*^2^ value of all dependent variables in the path model is exceeded zero (>0). In this study, the *Q*^2^ values in Table 4 are all above zero, so all dependent variables in the path model have predictive relevance.

**Table 4:** Blindfolding statistics for predictive relevance (*Q*^2^) for the general model.

Constructs	SSO	SSE	*Q*^2^ (= 1−SSE/SSO)
Customer directed OCB	1,460	1360.614	0.068
Innovative work behavior	1,095	982.278	0.103
Team selling performance	1,460	1160.597	0.205
Transactive memory system	3,285	3240.187	0.014

*N* = 365; SSO, sum of the square of observation; SSE, sum of the square of prediction error; and OCB, organizational citizenship behavior.

### Significance of Path Coefficient

Table 5 shows the results of path relationships in the proposed model. Results demonstrate that authentic sales leadership positively relates to the TMS (*β =* 0.176, *p =* 0.001). Hence, H1 supporting the research. Furthermore, the results of the hypotheses suggest that authentic sales leadership has a statistically significant and positive effect on innovative work behavior (*β =* 0.514, *p =* 0.000), supporting H2. Similarly, authentic sales leadership is positively and significantly related to customer-directed OCB (*β =* 0.361, *p =* 0.000). Thus, H3 is supported. For H4a and H4b, results show that TMS is significantly and positively associated with innovative work behavior (*β =* 0.481, *p =* 0.026) and team selling performance (*β =* 0.253, *p =* 0.002). Moreover, for H5a and H5b, the results of the hypothesis suggest that customer-directed OCB is significantly and positively associated with innovative work behavior (*β =* 0.280, *p =* 0.000) and team selling performance (*β =* 0.347, *p =* 0.000), supporting H5a and H5b. In last, the positive outcomes could be seen among the linkage between innovative work behavior and team selling performance (*β =* 0.323, *p =* 0.010). Hence, H6 supported the study.

**Table 5:** Hypotheses testing.

Hypotheses		*β*	*p*-value	*t*-value	CIs		Sig. <0.05
					2.50%	97.50%	
H1	AL → TMS	0.176	0.001	3.212	0.075	0.307	Supported
H2	AL → IWB	0.514	0.000	7.427	0.351	0.628	Supported
H3	AL → CDOCB	0.361	0.000	6.379	0.250	0.469	Supported
H4a	TMS → IWB	0.481	0.026	4.574	0.040	0.249	Supported
H4b	TMS → TSP	0.253	0.002	2.179	0.016	0.487	Supported
H5a	CDOCB → IWB	0.280	0.000	0.116	0.088	0.348	Supported
H5b	CDOCB → TSP	0.347	0.000	5.577	0.120	0.395	Supported
H6	IWB → TSP	0.323	0.010	1.850	0.082	0.716	Supported
H7	CDOCB × IWB → TSP	0.081	0.012	0.102	0.014	0.120	Supported
H8a	AL → TMS → TSP	0.045	0.157	1.419	−0.111	0.012	Not Supported
H8b	A → IWB → TSP	0.166	0.009	2.267	0.038	0.265	Supported
H8c	AL → CDOCB → TSP	0.125^*^	0.000	4.457	0.046	0.156	Supported

*N* = 365; CI, confidence interval; AL, authentic leadership; TMS, transactive memory system; IWB, innovative work behavior; CDOCB, customer-directed extra role behavior; and TSP, team selling performance.

### Findings of Moderation and Mediation

To test H7 regarding the moderating role of customer-directed OCB, we executed the moderation regression analysis to examine the interaction effect of customer-directed OCB between authentic leadership behavior and team selling performance relationship. The interaction term was added in the model to see its impact on the relationship. As we hypothesized, the findings exhibit a positive and significant moderating effect of customer-directed organizational citizenship behavior (*β* = 0.081, *p* = 0.012). It means the effect of innovative work behavior on team selling performance will be stronger when salespeople show their extra-role behaviors toward customers.

**Figure 3:**
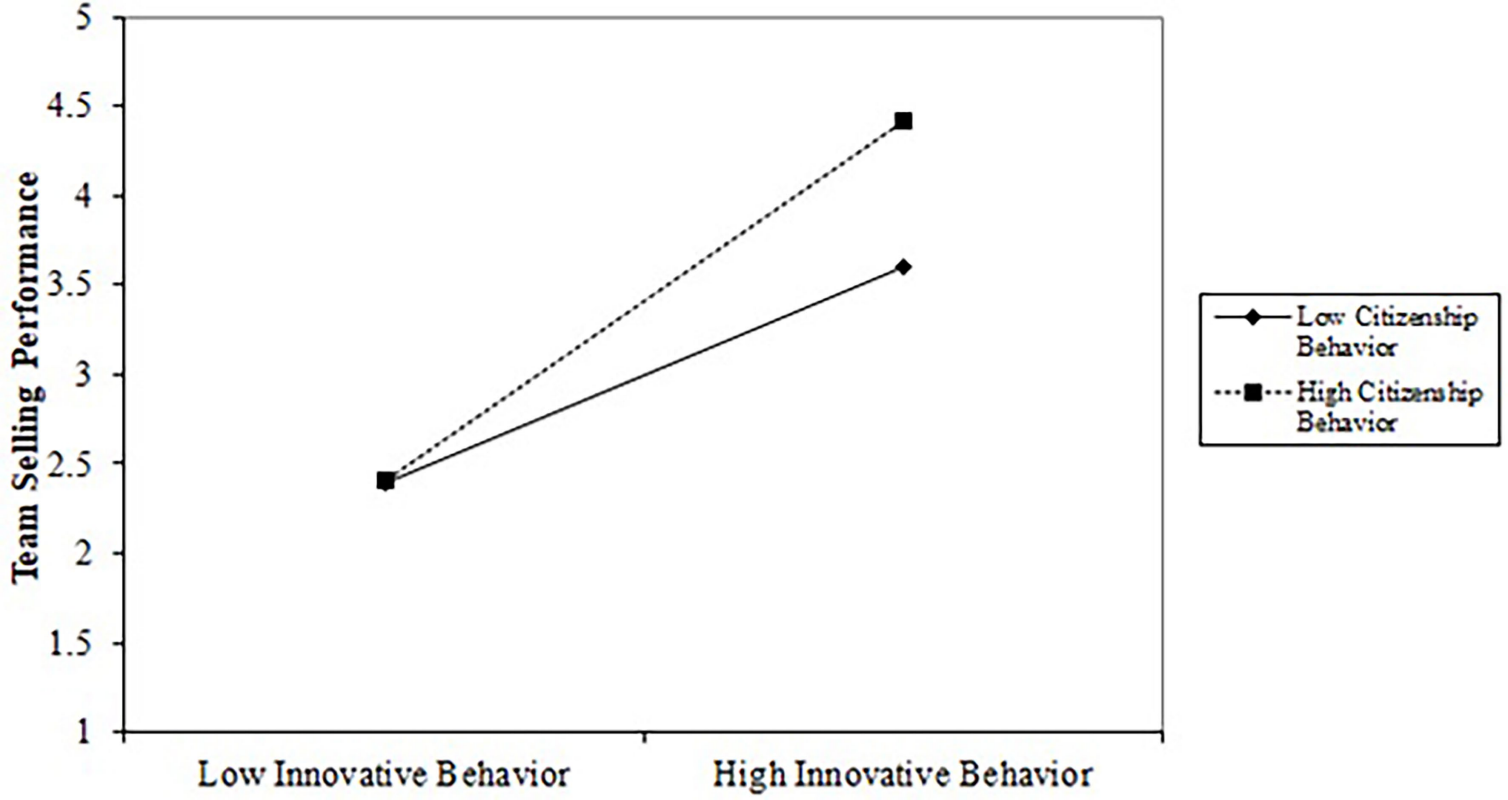
Simple slope analysis.

We also measured the implication of mediating variables in the model. Table 5 and Figure 3 show that innovative work behavior (*β =* 0.166, *p =* 0.009) and customer-directed citizenship behavior (*β =* 0.125, *p =* 0.000) significantly mediate the relationship between authentic sales leadership and team selling performance. Thus, H8b and H8c fully supported the study. However, the TMS is not significantly mediate between the relationship of authentic sales leadership and team selling performance.

## Discussion

The primary goal of this empirical research was to establish and validate a research model intended to improve the understanding of sales leadership style toward team-related outcomes in sales organizations. To this purpose, we have established a conceptual model and examined the outcomes of sales managers’ authentic leadership in the evolution of TMSs among salespeople in the B2B context. We also investigated innovative work behavior and customer-directed OCB as mediation channels between authentic leadership and team selling performance relationships.

With an emphasis on the COR theory, our examination facilitates a preliminary overview of the theoretical gap by connecting authentic leadership style to the sales employees’ customer-directed OCB. Sales leaders can operate as the origin of resources (Braun and Nieberle, 2017), which can be used by salespeople to develop a positive resource strategy to obtain additional resources, experience spirals of resource acquisition, and invest their resources in behaviors above the job requirements (Halbesleben et al., 2014), such as customer-directed OCB and employee innovation. The research reveals that sales managers’ authentic leadership encourages the subordinates’ to engage in discretionary behaviors and boost confidence to serve customers above and beyond their minimum expectations. The study shows a positive nexus among sales managers’ authentic leadership and customer-directed OCB in the B2B sales context, and the findings are parallel with the study of MacKenzie et al. (1993) and Luu (2020). These findings have claimed that authentic leaders will impact salespeople to satisfy their customers in terms of extra-role behavior. The supplementary findings further investigated the mediation mechanism of innovative work behavior for the association between authentic sales leadership and team selling performance. However, innovative work behavior is often considered impulsive and beyond the obligation of the salesperson. Therefore, sales managers must also have an appropriate atmosphere for trust, belief, and interest in these innovative functions (Anderson et al., 2004). This mediation mechanism is not only aligned with a resource-based view of authentic leadership (Braun and Nieberle, 2017) but still in line with the recent study focused on mediation process (i.e., behaviors that creates better performance) adaptive selling behavior (Wong et al., 2015) and counterproductive behavior. The implications of this research give a clearer overview, including its outcomes of how sales managers’ authentic leadership, directly and indirectly, influences internal & external behaviors of sales employees and the overall team performance.

### Research Implication

This research adds to the sales literature in multiple courses of action. By following the principle of COR as a theoretical foundation, we have introduced and analyzed a conceptual model that highlights authentic leadership style as a resource for salespeople in retrieving and sharing useful knowledge among teams. Previous literature has focused on the concept of a TMS in different leadership domains such as shared leadership (Ong et al., 2020) and knowledge leadership (Zhang and Guo, 2019). However, this study shows that authentic leadership style is a vital situational predictor of a TMS in the B2B sales context, which has been largely neglected by the previous scholars. To build trust, authentic leaders should encourage their subordinates to believe in the workplace environment and be able to retrieve and exchange useful knowledge with other colleagues (Peterson et al., 2012). Leadership literature focuses on the role of leaders in team achievement (Morgeson et al., 2010). In order to strengthen the focus on teams to address the obstacles for improving employees’ knowledge requirement, the managers need insights into which various types of leadership styles are more useful when using TMS to enhance team performance (i.e., authentic leadership). Besides, our results propose that managers engaged in authentic sales leadership leads to a greater level of TMS and these findings are consistent with the study of Bachrach and Mullins (2019), who found a positive association between leadership behavior and TMS. This might have been an efficient platform for the development practices that enhances knowledge efforts and team overall performance.

### Managerial Implication

This study has many implications for sales corporations and leaders. Managers should utilize their expertise to address successful TMS generation by implementing team behavioral traits that have the potential to affect the performance of different leadership styles. An authentic sales leadership seems to be a more productive strategy for producing TMS when sales teams are smaller, which would benefit overall team performance. Our study shows logical ways to manage team structures and advise managers who encourage information sharing and teamwork activities among their salespeople in order to enhance each team member’s intrinsic knowledge for overall performance. Managers can inspire salespeople to develop and exchange their TMS-specific expertise.

Furthermore, sales managers can train and encourage their employees, during which they can use additional job-related resources (knowledge, skills) to engage in customer-directed OCB effectively. Organizations can set the picture for more successful use of authentic leadership style in the development of innovative work behavior. Authentic leaders must consider the execution of innovative strategies through a series of conferences, training sessions, social events, and friendly competitions, to fostering the emotional intelligence of salespeople and for the overall organizational innovation capability. Overall, the study found positive association of a supportive relationship between authentic sales leadership and innovative work behavior and has revealed that authentic leadership behaviors in sales managers will promote strategic engagement and creative performance.

### Limitations and Future Research

In light of our research observations, several new opportunities for future studies are recognized. The present research investigates the consequence of authentic sales leadership on the salesperson’s behavioral antecedents. In the future, researchers could enlarge the model by adding a salesforce control system (i.e., behavior-based control and outcome-based control) as an exogenous variable in replacement for authentic sales leadership. Furthermore, many other researchers conclusively indicated three sub-dimensions of TMS (Liang et al., 1995). Consequently, our research addressed TMS as a one-dimensional paradigm, which is also the limitation of this study. Future research should investigate how authentic sales leadership influences different dimensions of TMS (specialization, coordination, and credibility).

We performed this study by conducting a survey at a single time frame, and then we matched survey responses with selling performance data provided by the sales managers. So, it would be interesting to take longitudinal data allowing the researcher to assess changes over time. In Figure 2, the explained variance for team selling performance is (*R*^2^ = 76.6%), which is above 10% of the threshold recommended by Falk and Miller (1992). This indicates that any other endogenous variables should be introduced to improve the predictive strength of team selling performance such as personal selling strategies (e.g., adaptive selling, up-selling or cross-selling) are treated as predominant indicators in team selling performance when employing sales leadership behaviors (Johnson and Friend, 2015; Singh et al., 2017). The study involves control variables based on the salesperson’s demographics, and the future research may employ other control variables in the pharmaceutical context, i.e., (1) typology of the firms according to the business model and strategy formulation (2) typology of products, customers, and channels. There is another limitation involve in this study, the choice of a single geographical background as a target population. So in the future, data should be obtained from various metropolitan areas for the consistency of results and its generalization.

## Data Availability Statement

The raw data supporting the conclusions of this article will be made available by the authors, without undue reservation.

## Author Contributions

MS wrote the major part of the manuscript and contributed to data collection and data analysis. The introduction, literature review, and methodology sections are written and revised by TI and KB. In addition, MA greatly improved the data analysis and interpretation of the study. The final draft has been proofread and approved by BA. All authors contributed to the article and approved the submitted version.

## Conflict of Interest

The authors declare that the research was conducted in the absence of any commercial or financial relationships that could be construed as a potential conflict of interest.

## Publisher’s Note

All claims expressed in this article are solely those of the authors and do not necessarily represent those of their affiliated organizations, or those of the publisher, the editors and the reviewers. Any product that may be evaluated in this article, or claim that may be made by its manufacturer, is not guaranteed or endorsed by the publisher.
